# Supplementary material for: Kilohertz electrical stimulation drives widespread neuronal activation and temporal interference imposes focal rhythmic modulation
Source: bioRxiv. 2026 Oct 21:2026.10.15.759497. Preprint. [Version 2] doi: 10.64898/2026.10.15.759497 (PMC13738535; doi:10.64898/2026.10.15.759497)
Supplement: Supplementary file 1 [file NIHPP2026.10.15.759497v2-supplement-1.pdf]

bioRxiv preprint doi: https://doi.org/10.64898/2026.10.15.759497; this version posted October 21, 2026. The copyright holder for this preprint (which was not certified by peer review) is the author/funder, who has granted bioRxiv a license to display the preprint in perpetuity. It is made available under a CC-BY 4.0 International license.

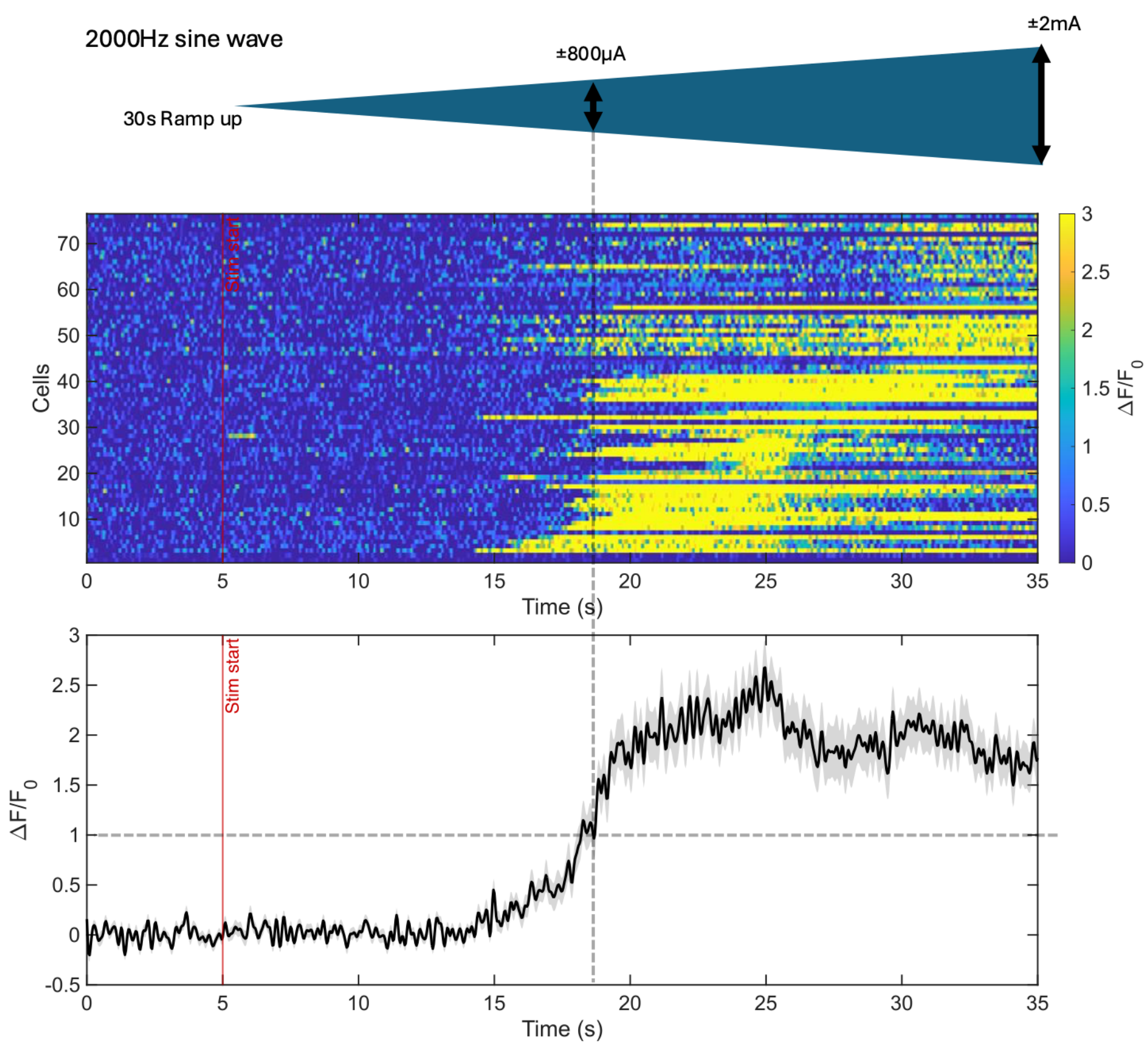

Supplementary Fig. 1. Neurons showed responses during long-ramp-up stimulation regardless of the gradually increasing stimulus amplitude.
After a 5-s baseline, 2000Hz sine wave stimulation amplitude was linearly ramped for 30-s. Top, schematic of the long-ramp-up stimulation waveform, increasing linearly from 0 to ±2 mA over

bioRxiv preprint doi: https://doi.org/10.64898/2026.10.15.759497; this version posted October 21, 2026. The copyright holder for this preprint (which was not certified by peer review) is the author/funder, who has granted bioRxiv a license to display the preprint in perpetuity. It is made available under a CC-BY 4.0 International license.

30-s. Middle, $\Delta F/F_0$ heatmap for cells (n = 76). Color indicates $\Delta F/F_0$. Bottom, population mean $\Delta F/F_0$ across all cells (black); shaded area indicates SEM.

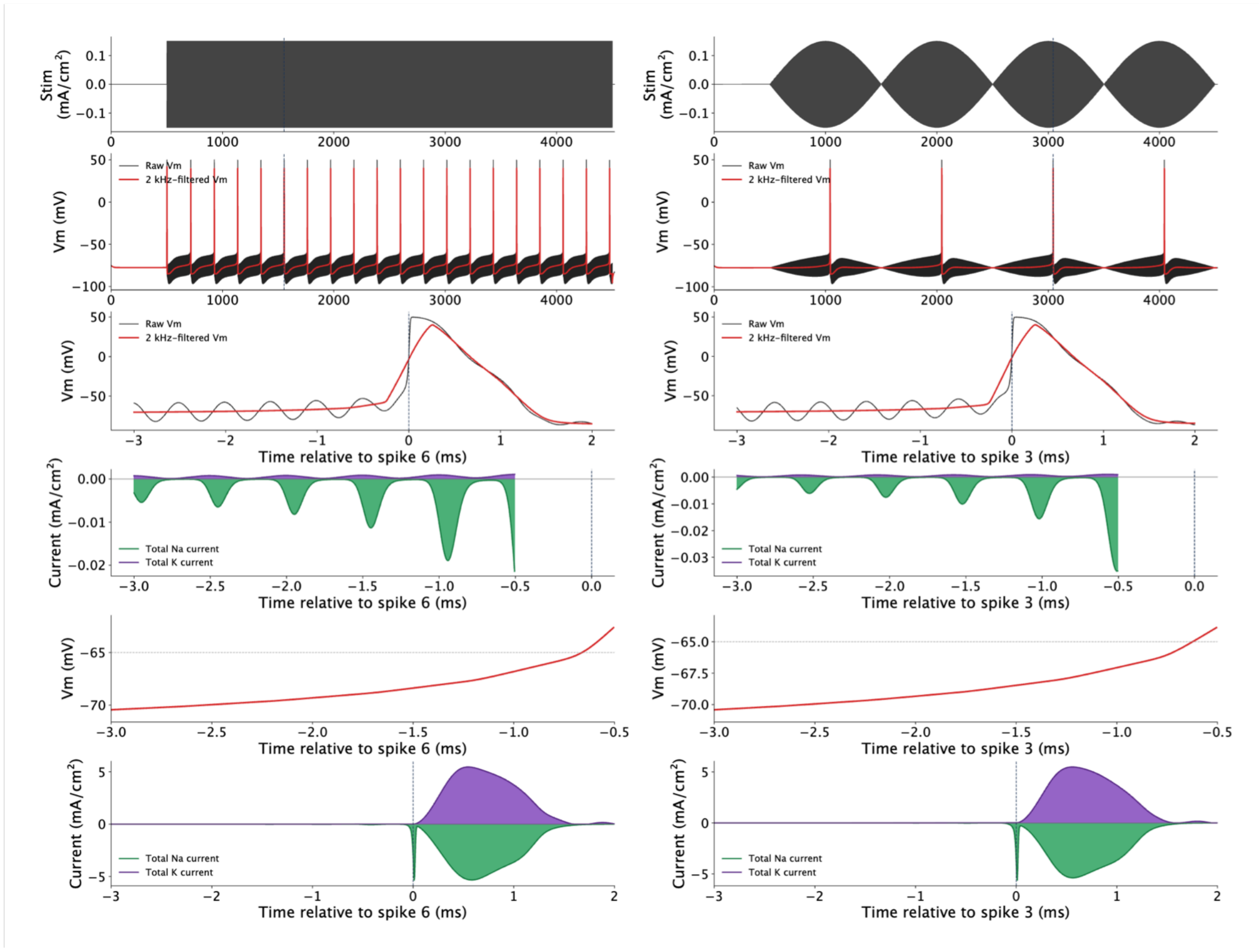

Supplementary Fig. 2. Simulated responses of Reduced single-compartment axonal model to high-frequency stimulation.
Left, reduced single-compartment axonal model under unmodulated carrier stimulation. Right, reduced single-compartment axonal model under TI stimulation. From top to bottom, traces show: (1) the applied stimulation waveform; (2) membrane potential over the full stimulation period, with the 2-kHz-filtered membrane potential shown in red; (3) membrane potential immediately preceding and during spike initiation; (4) sodium and potassium currents during the corresponding pre-spike period; (5) the filtered membrane potential during the subthreshold period preceding spike initiation; and (6) the corresponding filtered sodium and potassium currents. Green indicates inward sodium current and purple indicates outward potassium current.

bioRxiv preprint doi: https://doi.org/10.64898/2026.10.15.759497; this version posted October 21, 2026. The copyright holder for this preprint (which was not certified by peer review) is the author/funder, who has granted bioRxiv a license to display the preprint in perpetuity. It is made available under a CC-BY 4.0 International license.

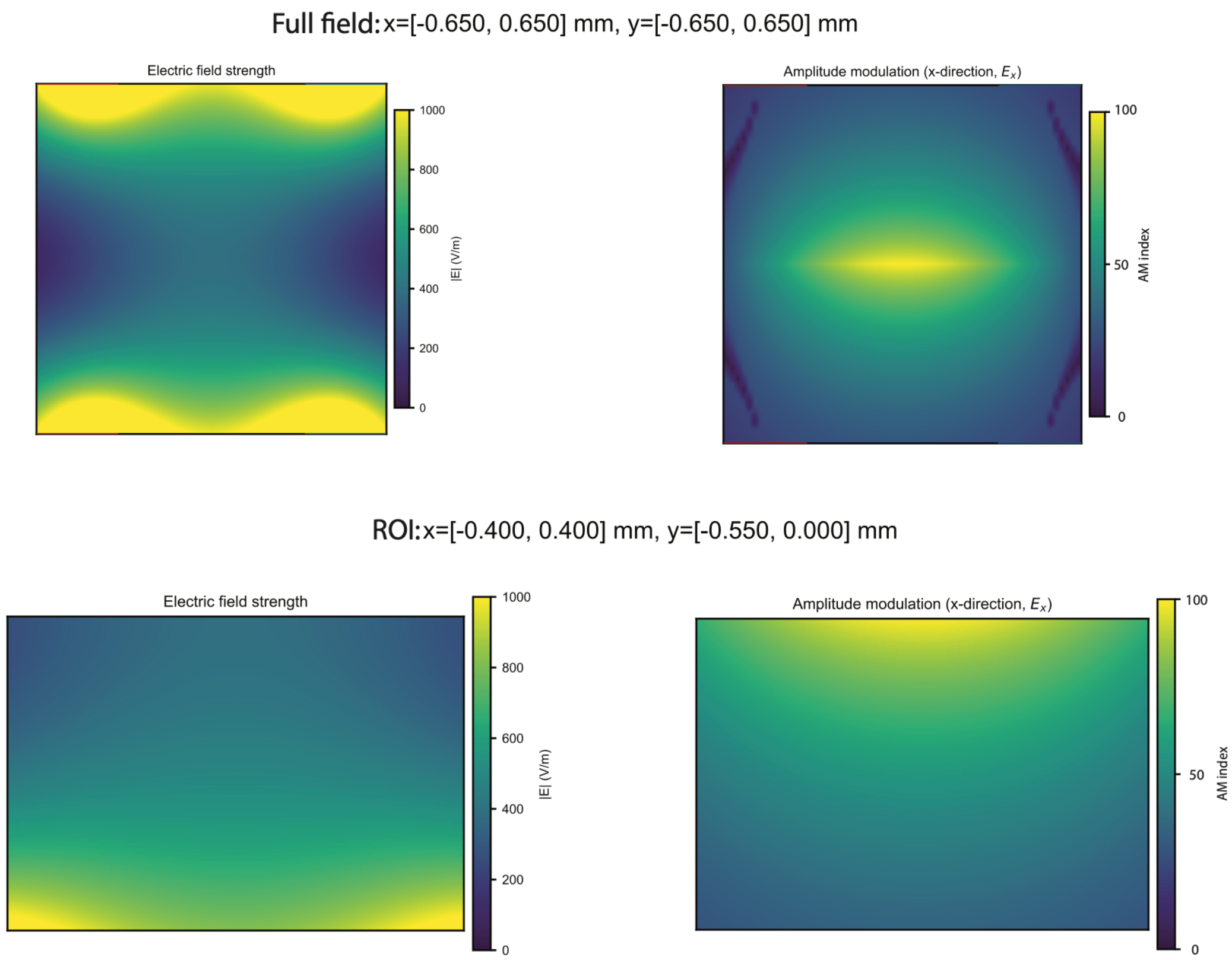

Supplementary Fig. 3. Simulated electric field and AM index for the conventional TI electrode configuration *ex vivo*

Left panels show the electric-field magnitude (|E|), and right panels show the normalized AM index of the x-component of the electric field (Ex). The top row shows the full field distribution (x = −0.65 to 0.65 mm, y = −0.65 to 0.65 mm), whereas the bottom row shows the region of interest (ROI; x = −0.40 to 0.40 mm, y = −0.55 to 0 mm) used for spatial analysis. The two electrode pairs were positioned at y = −0.65 and 0.65 mm, respectively, with the electrodes within each pair located at x = −0.5 and 0.5 mm. Simulations were performed with a 1-mm intra-pair electrode spacing, a 1.3-mm inter-pair distance, a 2-mm exposed electrode length, a uniform conductivity of 0.6 S/m, and a stimulation amplitude of 4 mA per electrode.

bioRxiv preprint doi: https://doi.org/10.64898/2026.10.15.759497; this version posted October 21, 2026. The copyright holder for this preprint (which was not certified by peer review) is the author/funder, who has granted bioRxiv a license to display the preprint in perpetuity. It is made available under a CC-BY 4.0 International license.

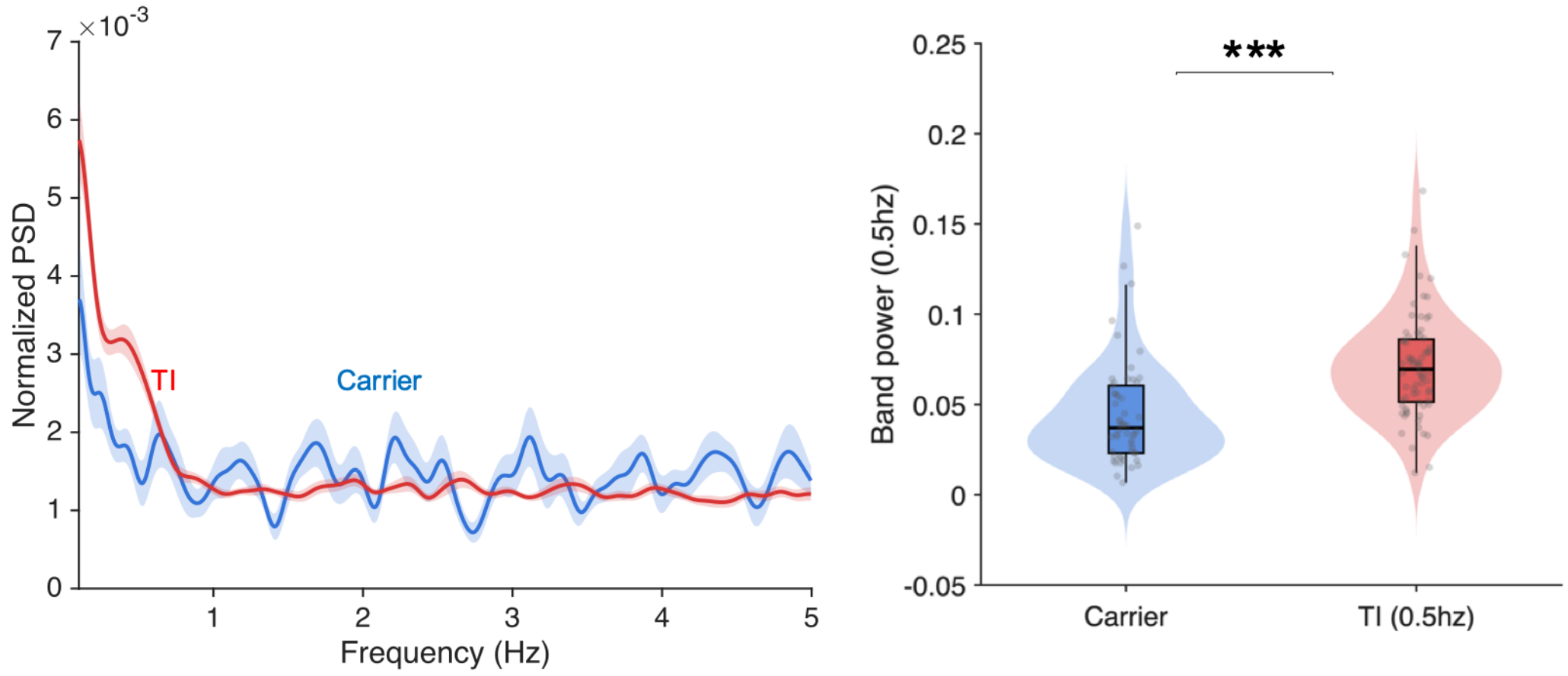

Supplementary Fig. 4. 0.5-Hz TI stimulation enhances neuronal entrainment at the beat frequency relative to carrier stimulation.

Comparison of normalized PSD power in responsive neurons during carrier and TI (0.5-Hz) stimulation. Left, normalized PSD spectra from 0.1 to 5-Hz for carrier (2-kHz; blue) and TI (0.5-Hz; red) stimulation. Thick traces indicate the mean and shaded areas indicate SEM across responsive neurons; the dashed line marks 0.5-Hz. Right, quantification of normalized 0.5-Hz band power (0.4–0.6-Hz). Violin plots show the distribution for each condition; box plots indicate the median and interquartile range, and dots indicate individual cells. LMM, $p < 0.05$; n = 72 cells for TI and n = 54 cells for carrier from 3 mice.

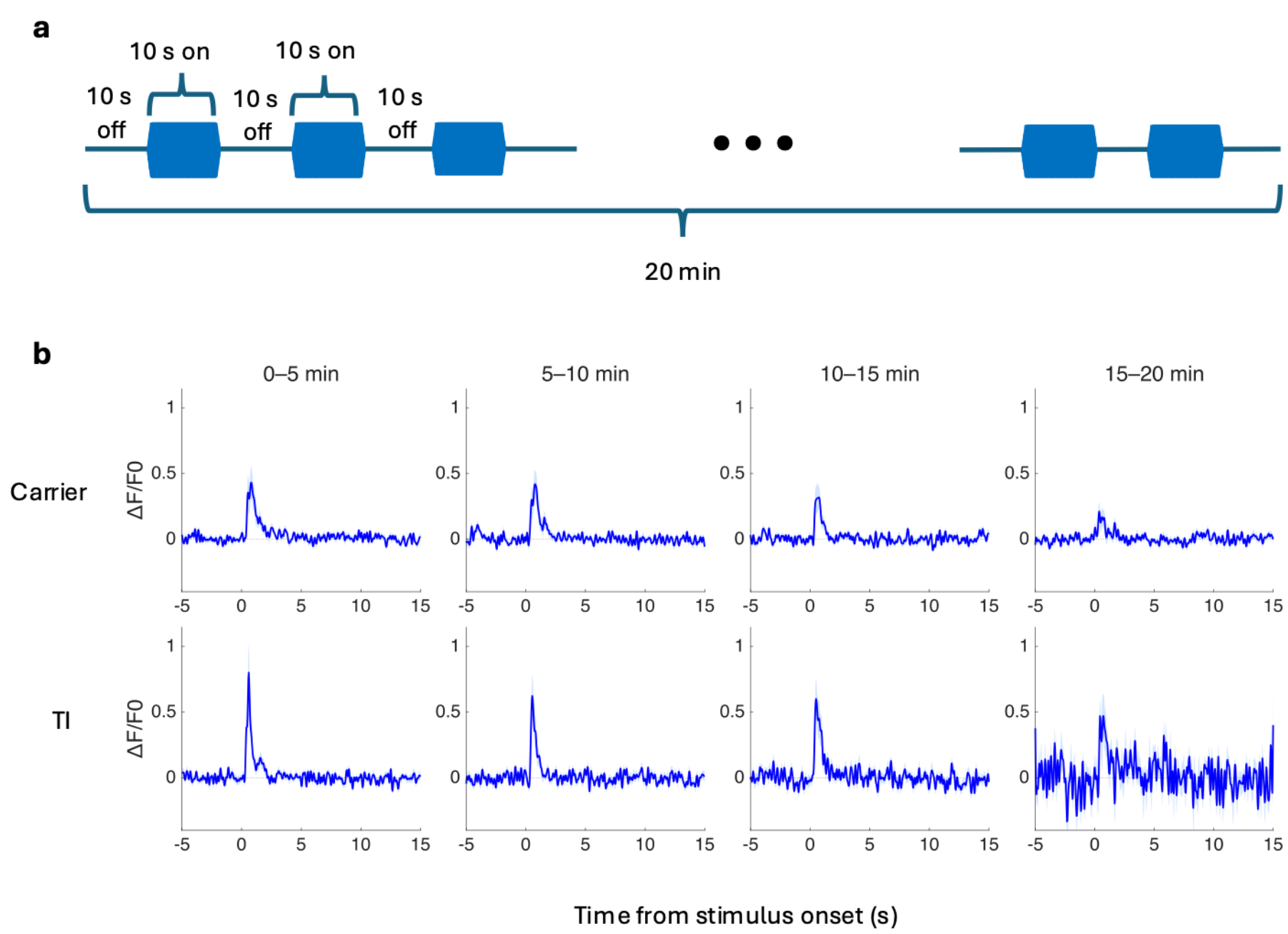

Supplementary Fig. 5. Sustained calcium responses during 20-min carrier and TI stimulation.

a, stimulation paradigm: 10-s stimulation ON and 10-s OFF cycles were repeated for 20min. Carrier (2-kHz) or TI (2000 and 2010-Hz; 10-Hz beat frequency) was kept constant throughout each recording.

bioRxiv preprint doi: https://doi.org/10.64898/2026.10.15.759497; this version posted October 21, 2026. The copyright holder for this preprint (which was not certified by peer review) is the author/funder, who has granted bioRxiv a license to display the preprint in perpetuity. It is made available under a CC-BY 4.0 International license.

b, mean $\Delta F/F_0$ traces of all cells after stimulus onset, shown separately for four 5-min epochs (0–5, 5–10, 10–15, and 15–20-min). The upper row shows responses during carrier stimulation, and the lower row shows responses during TI stimulation. Data are from one mouse (28 cells).

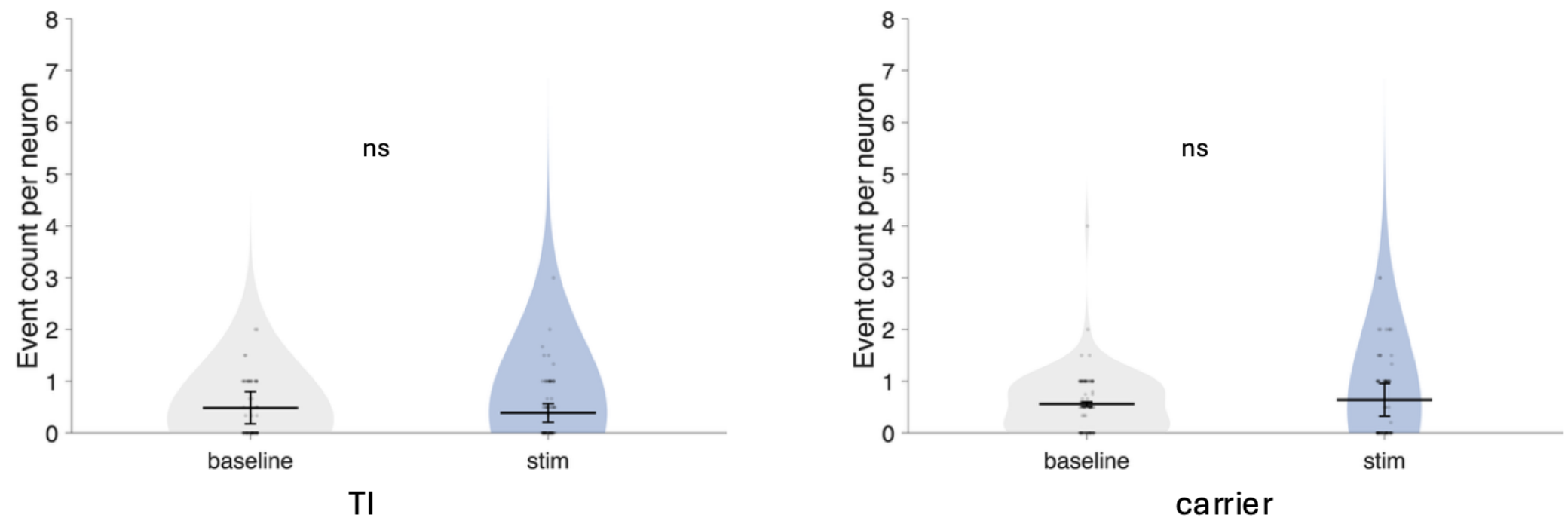

Supplementary Fig. 6. 250 uA did not induce a significant direct response
Event count per neuron during baseline (0-10 s) and stimulation (10-20 s) at 250 uA. Responsive ROIs were defined as neurons showing at least one detected event within 0-30 s in the 200 uA condition. Counts were extracted from traces and compared using a linear mixed-effects model. No significant increase in event count during stimulation was detected in either TI ($n = 78$ neurons, 3 mice, $p = 0.322$) or carrier ($n = 88$ neurons, 3 mice, $p = 0.773$).

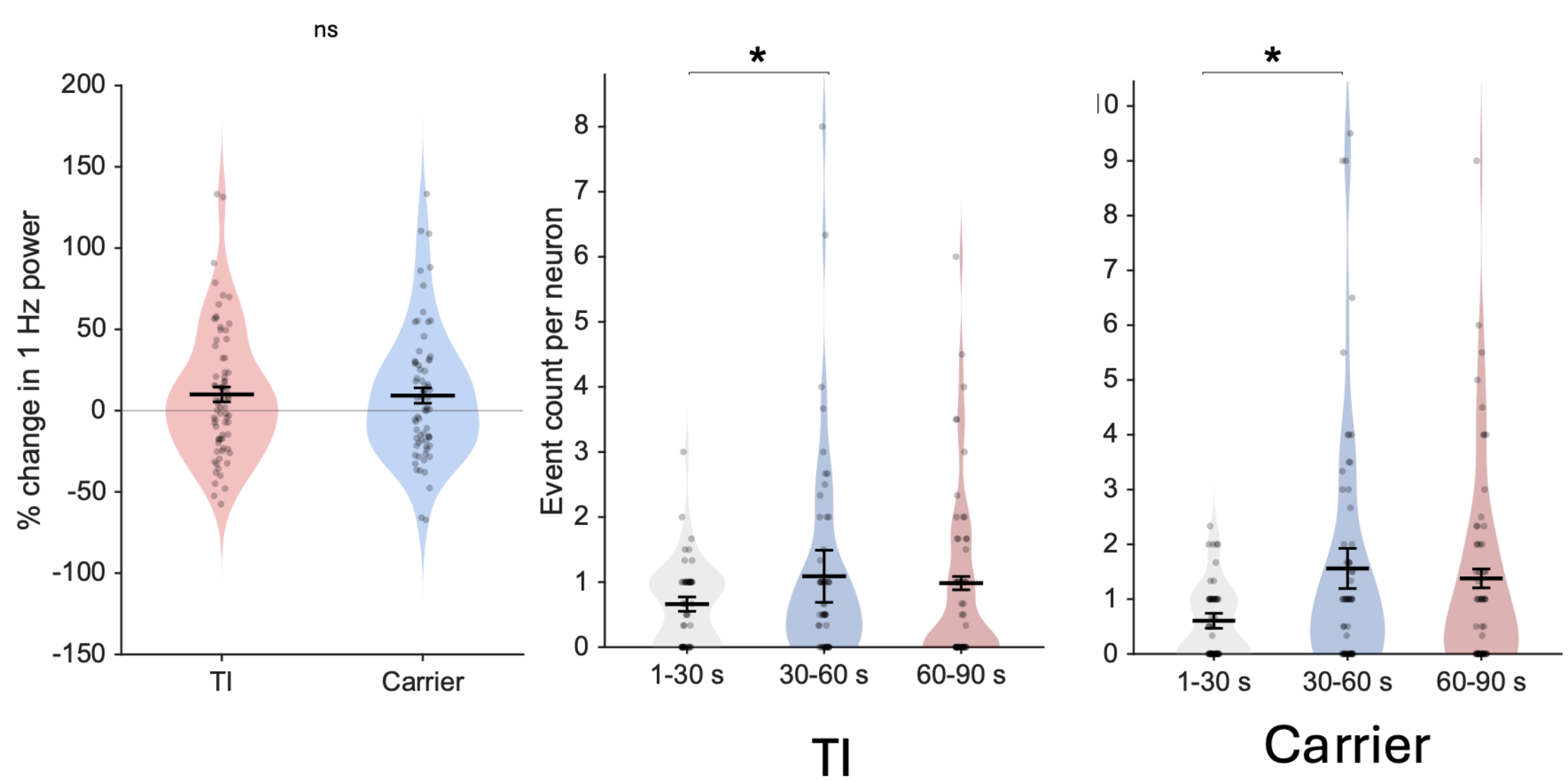

Supplementary Fig. 7. 5kHz carrier stimulation excited neuronal activity without a differential change in 1-Hz power
Left. Percent change in normalized 1-Hz power from the baseline period (0–30 s) to the stimulation period (30–60 s) in responsive neurons during TI and carrier (5 kHz) stimulation. Each dot represents one neuron; violins show distributions, and black bars indicate mean ± SEM. No significant difference was detected between TI and carrier stimulation (ns). Middle and Right. Event counts per responsive neuron during the 1–30 s, 30–60 s, and 60–90 s epochs for TI (Middle). and carrier (5 kHz) stimulation (Right), respectively. Each dot represents

bioRxiv preprint doi: https://doi.org/10.64898/2026.10.15.759497; this version posted October 21, 2026. The copyright holder for this preprint (which was not certified by peer review) is the author/funder, who has granted bioRxiv a license to display the preprint in perpetuity. It is made available under a CC-BY 4.0 International license.

one neuron; violins show distributions, and black bars indicate mean ± SEM. Statistical comparisons were performed using LMM. $p < 0.05$. n = 74 cells for TI, n = 71 cells for carrier from 3 mice
